# Serum Metabolic Signatures of Chronic Limb-Threatening Ischemia in Patients with Peripheral Artery Disease

**DOI:** 10.3390/jcm9061877

**Published:** 2020-06-16

**Authors:** Sandi M. Azab, Abdelrahman Zamzam, Muzammil H. Syed, Rawand Abdin, Mohammad Qadura, Philip Britz-McKibbin

**Affiliations:** 1 Department of Chemistry and Chemical Biology, McMaster University, Hamilton, ON L8S 4M1, Canada; azabs@mcmaster.ca; 2 Department of Pharmacognosy, Alexandria University, Alexandria 21521, Egypt; 3 Department of Surgery, St. Michael’s Hospital, Toronto, ON M5B 1W8, Canada; abdelrahman.zamzam@gmail.com (A.Z.); muzammil.syed@mail.utoronto.ca (M.H.S.); mohammad.qadura@utoronto.ca (M.Q.); 4 Department of Medicine, McMaster University, Hamilton, ON L8N 3Z5, Canada; rawand.abdin@medportal.ca

**Keywords:** peripheral artery disease, serum, metabolomics, intermittent claudication, chronic limb-threatening ischemia

## Abstract

Peripheral artery disease (PAD) is characterized by the atherosclerotic narrowing of lower limb vessels, leading to ischemic muscle pain in older persons. Some patients experience progression to advanced chronic limb-threatening ischemia (CLTI) with poor long-term survivorship. Herein, we performed serum metabolomics to reveal the mechanisms of PAD pathophysiology that may improve its diagnosis and prognosis to CLTI complementary to the ankle–brachial index (ABI) and clinical presentations. Non-targeted metabolite profiling of serum was performed by multisegment injection–capillary electrophoresis–mass spectrometry (MSI–CE–MS) from age and sex-matched, non-diabetic, PAD participants who were recruited and clinically stratified based on the Rutherford classification into CLTI (*n* = 18) and intermittent claudication (IC, *n* = 20). Compared to the non-PAD controls (*n* = 20), PAD patients had lower serum concentrations of creatine, histidine, lysine, oxoproline, monomethylarginine, as well as higher circulating phenylacetylglutamine (*p* < 0.05). Importantly, CLTI cases exhibited higher serum concentrations of carnitine, creatinine, cystine and trimethylamine-*N*-oxide along with lower circulating fatty acids relative to well matched IC patients. Most serum metabolites associated with PAD progression were also correlated with ABI (*r* = ±0.24−0.59, *p* < 0.05), whereas the ratio of stearic acid to carnitine, and arginine to propionylcarnitine differentiated CLTI from IC with good accuracy (*AUC* = 0.87, *p* = 4.0 × 10^−5^). This work provides new biochemical insights into PAD progression for the early detection and surveillance of high-risk patients who may require peripheral vascular intervention to prevent amputation and premature death.

## 1. Introduction

Peripheral artery disease (PAD) is a form of atherosclerosis that manifests in the lower extremities leading to a cascade of symptoms from insufficient blood flow, including impaired/painful walking, reduced functional capacity, ischemic myopathy, and recurrent skin lesions [1]. PAD is also associated with higher risk for cardiovascular events such as myocardial infarction, stroke, and vascular death [2]. Although most PAD patients are asymptomatic, patients with PAD usually present with intermittent claudication (IC), characterized with varying degrees of pain in leg muscles induced by walking. If left untreated, patients can progress to a severe end-stage form of PAD known as chronic limb-threatening ischemia (CLTI), which is characterized by rest pain, non-healing ischemic ulcers, and gangrene requiring limb amputation [2]. Disease progression in PAD is highly variable and unpredictable as some CLTI patients who undergo amputations do not exhibit any PAD symptoms 6 months before onset [3]. In fact, cardiovascular events are more prevalent among CLTI patients than coronary artery disease (CAD) indicating significant associated morbidity [3]. Although CLTI accounts for less than 5% of total PAD diagnoses, survivorship is poor with a 5-year mortality rate of about 50% [4]. As a result, there is an urgent need for understanding the mechanisms of PAD progression for the early detection of CLTI that also guides evidence-based treatment decisions [5].

Despite a high estimated prevalence of 10–20% among older persons, PAD is often undiagnosed and/or untreated in the primary care setting with most physicians unaware of the diagnosis [6]. In practice, diagnosis is confirmed in symptomatic patients by an abnormal resting ankle–brachial index (ABI) below 0.90, which is determined by the ratio of the systolic blood pressure at the ankle of the affected leg to the upper arm, using a Doppler ultrasound blood flow detector [1]. Usually, ABI is a reliable diagnostic tool for PAD diagnosis that may also predict atherosclerotic PAD mortality when performed in accredited laboratories with specialized equipment and training [7]. However, in 25% of diabetic patients, ABI lacks sensitivity to diagnose patients with PAD due to peripheral arterial stiffening from calcification [8]. Moreover, the benefit of the routine screening of PAD risk in asymptomatic patients using ABI remains inconclusive based on the recent findings from the US Preventive Services Task Force [9]. For these reasons, specific yet sensitive blood-based biomarkers are needed for PAD diagnosis and risk assessment that is applicable for routine testing in a clinical setting, including the surveillance of high-risk CLTI patients following revascularization interventions.

Metabolomics offers a systemic approach for the molecular phenotyping of complex biological processes underlying cardiovascular diseases (CVD) as required for new advances in precision medicine and drug development [10]. Comprehensive metabolite profiling using high-field nuclear magnetic resonance (NMR) and increasingly high-resolution mass spectrometry (MS) enables the discovery of clinically relevant biomarkers associated with atherosclerosis, that reflect the dynamic interactions between the host, gut microbiota, and dietary exposures, such as trimethylamine-*N*-oxide [11]. Growing evidence also demonstrates that elevated plasma branched-chain amino acid concentrations increase the risk for stroke that may be counteracted by Prudent diet modifications [12]. However, most metabolomic studies to date have focused on identifying aberrant metabolic pathways in CAD as compared to standard predictors [13]. In contrast, there have been few reports using metabolomics to understand the pathophysiology of PAD, which is prone to confounding since older patients often suffer from other comorbidities, including type 2 diabetes, chronic kidney disease and/or cardiovascular events [14,15,16,17]. Herein, we apply an untargeted serum metabolomics data workflow with stringent quality control (QC) on a clinically stratified non-diabetic patient cohort for the assessment of PAD progression that may allow for better therapeutic monitoring of patients as compared to conventional ABI and clinical assessment tools.

## 2. Materials and Methods

### 2.1. Study Cohort and Design

This study was approved by the research ethics board at St. Michael’s Hospital-University of Toronto (REB #16-375), and informed consent was obtained from all participants in accordance with the Declaration of Helsinki principles. PAD diagnosis and classification into IC and CLTI were made according to specialists’ clinical examination and arterial ultrasound [18]. Patients with CLTI or “Rutherford stage ≥ 4” referred to vascular surgery ambulatory clinics or emergency department at St. Michael’s Hospital (Toronto, ON, Canada) from June 2017 to August 2017, were requested to participate in this study. Exclusion criteria included all patients on anticoagulants, chemotherapy or biological anti-inflammatory agents. Patients diagnosed with sepsis, type 2 diabetes, systematic inflammatory disease or with an active/history of any cancer or deep vein thrombosis were excluded as well. Moreover, patients with a 6-month history of acute coronary syndrome, heart failure, or uncontrolled arrhythmia, as defined by the American College of Cardiology, also failed to meet the inclusion criteria of this study [19]. A total of 128 consecutive ambulatory patients were initially recruited, however only 20 CLTI patients met the inclusion criteria and consented for this study. Upon acceptance to participate, CLTI patients were matched with non-diabetic IC cases or “Rutherford stage 1–3”, and non-PAD controls in a ratio of 1:1:1 by age group and biological sex. To do so, during the months of January and February 2018, 20 IC patients and 20 non-PAD participants were recruited to match the CLTI cohort. PAD status was defined clinically as per the Rutherford classification, whereas non-PAD controls were defined as patients with cardiovascular risk factors alongside a normal arterial ultrasound of the lower limbs, and palpable distal pulses without a significant clinical history of claudication. However, after matching our cohorts, two CLTI patients later withdrew their consent and were not included in the final study.

### 2.2. Baseline Patient Clinical Assessments

Medical history included details of any previous acute coronary syndrome, hyperlipidemia, arterial arrhythmia, arterial hypertension, renal disease, congestive heart failure, history of stroke or transient ischemic attack, history of cancer, diabetes, and smoking status. Hyperlipidemia was defined as total cholesterol > 5.2 mM or the use of anti-hyperlipidemic medication. Hypertension was defined as systolic blood pressure ≥ 130 mmHg or diastolic pressure ≥ 80 mm Hg, or the use of antihypertensive medication. Renal disease was defined as an estimated glomerular filtration rate of less than 60 mL/min/1.73 m^2^ as per the Kidney Disease Outcomes Quality Initiative 2002 guidelines, since serum cystatin C and urinary creatinine clearance are not measured routinely for vascular patients. Diabetes mellitus was defined as glycosylated hemoglobin A1c ≥ 6.5% or the use of antidiabetic medication. Smoking status was recorded for each patient.

### 2.3. Chemicals and Reagents

All chemicals were purchased from Sigma Aldrich (St. Louis, MO, USA) unless otherwise stated. Stock solutions for internal standards and metabolite standards were prepared in deionized water from a Barnstead EASYpure^®^ II LF system (Dubuque, IA, USA) for hydrophilic metabolites, and in methyl-*tert*-butyl ether (MTBE) for lipophilic fatty acids. Ultra-grade LC–MS solvents (acetonitrile, methanol, 2-isopropanol and water) purchased from Caledon Laboratories Ltd. (Georgetown, ON, Canada) were used to prepare sheath liquid solution for spray formation and aqueous or nonaqueous background electrolyte (BGE) for the capillary electrophoresis (CE) separations. All stock solutions for chemical standards were stored at 4 °C. Nanosep^®^ 3k Omega^TM^ ultrafiltration devices (Pall Life Sciences, Port Washington, NY, USA) were used for processing the diluted serum samples with a 3 kDa molecular weight cut-off filter for protein removal.

### 2.4. Serum Creatinine Measurement by Jaffé Method

Measurement of serum creatinine was performed at St. Michael’s Hospital using a modified Jaffé colorimetric assay on a Beckman Coulter AU system/analyzer (Beckman Coulter, Inc., Brea, CA, USA). Briefly, creatinine standard reagent (OSR6678) was added to an aliquot of serum, where the serum creatinine reacted with picric acid under alkaline conditions to form a yellow-orange complex and the rate of change in absorbance at 520/800 nm was proportional to the serum creatinine concentration to minimize other reacting/absorbing interferences [20]. Serum creatinine measurements by the Jaffé colorimetric assay were compared with multisegment injection–capillary electrophoresis–mass spectrometry (MSI–CE–MS) using independent serum aliquots collected from the same participants.

### 2.5. Serum Sample Collection and Preparation

Fasting blood samples were collected and immediately centrifuged at 4 °C within 1 h after clotting at room temperature, where the serum was separated, aliquoted and stored frozen at −80 °C. Frozen serum was then thawed slowly on ice, vortexed for 30 s and aliquoted prior to ultrafiltration or liquid extraction. An aliquot of 50 μL of serum was diluted two-fold with ultra-grade LC–MS water containing 40 μM of two recovery standards, 3-chloro-*L*-tyrosine (Cl-Tyr) and 3-cyclohexylamino-1-propanesulfonic acid (CAPS). The diluted serum was vortexed for 30 s, transferred to a pre-rinsed ultrafiltration device, that was centrifuged at 14,000× *g* for 10 min to separate the proteins from the serum filtrate used for the analysis of polar/hydrophilic metabolites. Ultrafiltration devices were pre-rinsed with ultra-grade LC–MS water, centrifuged for 5 min at 14,000× *g* and air dried for about 20 min prior to the first use to wash out background additives (e.g., lactic acid). Thereafter, serum filtrates (15 μL) were diluted one or two-fold with ultra-grade LC–MS water containing three internal standards, 4-fluoro-*L*-phenylalanine (F-Phe), 2-napthalenesulfonic acid (NMS) and ^13^C_6_-glucose. The final concentrations for all the internal and recovery standards were 10 μM with the exception of ^13^C_6_-glucose (2 mM), and all diluted serum filtrate samples were analyzed using MSI–CE–MS with two aqueous BGE systems optimal for the separation of ionic/hydrophilic metabolites [21,22].

A second aliquot of frozen serum was slowly thawed on ice and processed separately for lipid analysis following acid hydrolysis and liquid extraction as described previously [23,24]. In this work, the total (hydrolyzed) serum fatty acids were analyzed by multisegment injection–nonaqueous capillary electrophoresis–mass spectrometry (MSI–NACE–MS). Acid-catalyzed hydrolysis of esterified lipids was performed by the addition of 25 μL of serum, 25 μL of 2.5 M sulfuric acid and 25 μL of 0.01% vol butylated hydroxytouene (BHT) as an antioxidant additive in toluene followed by incubation at 80 °C for 1 h. Serum fatty acids were subsequently extracted using a slightly modified extraction protocol originally reported by Matyash et al. [25] using 500 μL of MTBE containing 50 μM of deuterated myristic acid (14:0-d27) as a recovery standard, with 12.5 μL of 1.0 M HCl for better extraction efficiency. Following vigorous shaking for 30 min at room temperature, phase separation was then induced by the addition of 100 μL of deionized water. Samples were then centrifuged at 3000× *g* at 4 °C to sediment the protein for 30 min resulting in phase separation into a water and ether (top) layer. A fixed volume (200 µL) was collected from the upper MTBE layer into a new vial then dried under a gentle stream of nitrogen gas at room temperature. Serum extracts were then stored dry at −80 °C and at the time of analysis reconstituted in 25 µL of acetonitrile/isopropanol/water (70:20:10 vol) with 10 mM ammonium acetate and 50 µM deuterated stearic acid (18:0-d35) as an internal standard. All hydrolysis reactions and extractions were carried out using glass GC vials that were pre-rinsed with dichloromethane and all the pipette tips used during this procedure were pre-rinsed with methanol to minimize the background palmitic acid (16:0) and stearic acid (18:0) contamination [23]. An internal quality control (QC) sample was prepared in-house by pooling together serum aliquots from all the study participants. Separate QC aliquots were processed using the same sample protocols described above, stored at −80 °C and thawed once prior to analysis by MSI–NACE–MS using a nonaqueous BGE system optimal for the separation of acidic lipids.

### 2.6. Hydrophilic Metabolome Profiling by MSI–CE–MS

MSI–CE–MS experiments were performed on an Agilent G7100A CE instrument (Agilent Technologies Inc., Mississauga, ON, Canada) equipped with a coaxial sheath liquid (Dual AJS ESI) Jetstream electrospray ionization source coupled to an Agilent 6550 quadrupole-time-of-flight (QTOF) system. An Agilent 1260 Infinity isocratic pump equipped with a 100:1 splitter and a 1260 Infinity degasser were used to deliver the sheath liquid at a rate of 10 μL/min. Separations were performed using uncoated fused-silica capillaries (Polymicro Technologies, Phoenix, AZ, USA) of a total length of 130 cm and an inner diameter of 50 μm. The BGE consisted of 1.0 M formic acid with 13% vol acetonitrile as the organic modifier (pH 1.8) under positive ion mode, and 35 mM ammonium acetate (pH 9.5) under negative ion mode for the comprehensive analysis of cationic and anionic serum metabolites, respectively [26,27,28]. Approximately 7 mm of the polyimide coating was removed from both the capillary inlet and the outlet using a capillary window maker (MicroSolv, Leland, NC, USA) to reduce the sample carry-over as well as the polymer swelling or degradation when in contact with organic solvent or buffer solutions containing ammonia [29,30]. Samples were injected hydrodynamically at 50 mbar (5 kPa) for 5 s for each sample, interspaced with alternating BGE spacer plugs injected hydrodynamically for 40 s (for positive ion mode) and electrokinetically for 45 s at 30 kV (for negative ion mode) for a total of 7 discrete serum filtrates analyzed within a single run. Customized serial injection configurations using calibrant mixtures, filtrate blanks, and pooled QC samples were analyzed in MSI–CE–MS depending on the study design requirements. Prior to first use, each bare fused-silica capillary was conditioned by flushing for 15 min at 950 mbar (95 kPa), sequentially, with methanol, 1.0 M sodium hydroxide, water, and BGE. An applied voltage of +30 kV at 25 °C was used for all the CE separations under normal polarity, however a pressure gradient of 2 mbar/min was implemented for the faster elution of slow migrating anions under negative ion mode conditions [29]. Between runs, the capillary was flushed with BGE for 15 min at 950 mbar (95 kPa). The sheath liquid was comprised of 60% vol MeOH with 0.1 vol% concentrated formic acid for positive ion mode, and 80% vol MeOH for negative ion mode detection. Reference ions purine and hexakis(2,2,3,3-tetrafluoropropoxy)phosphazine (HP-921) were spiked into the sheath liquid at 0.02% vol, providing constant mass signals to enable the real-time mass calibration and to allow for the monitoring of potential ion suppression effects during separation. The instrument was operated in a GHz extended dynamic range. The Vcap and nozzle voltage were both set at 2000 V, while the fragmentor was 380 V, the skimmer was 65 V and the octopole RF was 750 V. The QTOF–MS system was operated with full-scan data acquisition over a mass range of *m/z* 50–1700 and an acquisition rate of 1 spectrum/s. At the beginning of each day, the QTOF–MS system was calibrated before analysis using an Agilent tune mixture to ensure residual mass ranges did not exceed 1 ppm. Additionally, daily cleaning of the CE electrode and ion source with 50% vol isopropanol was performed as preventative maintenance. A standard mixture run followed by a QC sample run with blank were analyzed at the start of each day to equilibrate the CE–MS system and assess the system stability. Each serum filtrate sample from PAD and non-PAD participants in this study were analyzed in duplicate over two consecutive days by MSI–CE–MS under positive and negative ion modes for cationic/zwitter-ionic (e.g., amino acids) and anionic (e.g., organic acids) metabolites.

### 2.7. Lipophilic Metabolome Profiling by MSI–NACE–MS

Total serum (hydrolyzed) fatty acids were analyzed by MSI–NACE–MS under negative ion mode conditions as described elsewhere [23,24]. An Agilent 6230 time-of-flight (TOF) mass spectrometer with a coaxial sheath liquid electrospray (ESI) ionization source equipped with an Agilent G7100A CE unit was used for all experiments (Agilent Technologies Inc., Mississauga, ON, Canada). An Agilent 1260 Infinity Isocratic pump and a 1260 Infinity degasser were applied to deliver an 80:20 vol methanol:water with 0.5% vol ammonium hydroxide at a flow rate of 10 μL/min using a coaxial sheath liquid interface kit. For real-time mass correction, reference ions purine and hexakis(2,2,3,3-tetrafluoropropoxy)phosphazine (HP-921) were spiked into the sheath liquid at 0.02% vol to provide constant mass signals at *m/z* 119.0363 and *m/z* 1033.9881, which were also used to monitor for potential ion suppression/enhancement effects during separation. The nebulizer spray was set off during serial sample injection but then subsequently turned on at a low pressure of 4 psi (27.6 kPa), following voltage application with the source temperature at 300 °C and drying gas delivered at 4 L/min. The instrument was operated in a 2 GHz extended dynamic range with negative mode detection. Vcap was set at 3500 V while fragmentor was 120 V, the skimmer was 65 V and the Octopole RF was 750 V. Separations were performed on bare fused-silica capillaries with 50 μm internal diameter, 360 μm outer diameter and 95 cm total length (Polymicro Technologies Inc., Phoenix, AZ, USA), and a capillary window maker was used to remove about 7 mm of the polyimide coating on both ends of the capillary. The applied voltage was set to 30 kV at 25 °C for the CE separations together with an applied pressure of 20 mbar (2 kPa) used during the CE separation. The nonaqueous BGE was 35 mM ammonium acetate in 70% vol acetonitrile, 15% vol methanol, 10% deionized water, and 5% vol isopropanol with an apparent pH of 9.5 adjusted by the addition of 12% vol of ammonium hydroxide. Serum extracts, pooled QC extracts, or fatty acid calibrants were injected hydrodynamically at 50 mbar (5 kPa) alternating between 5 s for each sample plug and 40 s for the BGE spacer plug for a total of seven discrete samples analyzed within one run. Prior to the first use, the capillaries were conditioned by flushing for 15 min at 950 mbar (95 kPa) sequentially with methanol, 0.1 M sodium hydroxide, deionized water, 1.0 M formic acid, deionized water then BGE for 30 min. Between runs, the capillary was flushed with BGE for 10 min at 950 mbar (95 kPa), and nonaqueous BGE and sheath liquid solutions were degassed before use.

### 2.8. Data Processing and Statistical Analyses

All the MSI–CE–MS and MSI–NACE–MS data were analyzed using Agilent Mass Hunter Workstation Software (Qualitative Analysis, version B.06.00, Agilent Technologies Inc., Mississauga, ON, Canada). Molecular features were extracted in centroid and profile modes using a 10 ppm mass window for hydrophilic and lipophilic metabolites. Polar metabolites and hydrolyzed fatty acids were annotated based on their characteristic accurate mass (*m/z*) for their protonated, [M+H]^+^ or deprotonated, [M−H]^−^ molecular ion together with their relative migration time (RMT), where apparent migration times were normalized to an internal standard migrating from the same sample position [26]. Extracted ion electropherograms (EIEs) were integrated after smoothing using a quadratic/cubic Savitzky Golay function (15 points) and the peak areas and migration times were transferred to Excel (Microsoft Office, Redmond, WA, USA) for the calculation of relative integrated peak areas (RPAs) and relative migration times (RMTs). Data normalization to an internal standard (Cl-Tyr, CAPS, or 18:0-d35) improves the precision in CE by correcting for differences in sample volumes introduced in-capillary, as well as migration time drift due to the changes in electroosmotic flow between runs. Control charts for monitoring long-term method stability were derived from changes in measured responses (RPA) for recovery standards (F-Phe, NMS, or 14:0-d27) added to all the serum filtrates or extracts, including the QC samples. In most cases, serum metabolites and fatty acids from hydrolyzed lipids were identified with a high confidence (level 1) [31] using authentic standards with the exception of 8 metabolites (10% of total identified 85 metabolites; level 2) where standards were unavailable, as well as 7 unknown metabolites with only a putative molecular formula (level 4). Overall, 85 authentic metabolites were consistently detected (CV < 30%) in the majority of serum samples (>75%) when applying stringent quality control (QC) measures on 800 initially extracted features to filter redundant and spurious signals arising from contaminants, artifacts, isotopes, in-source fragments, adducts or dimers.

Least-squares linear regression analysis for external calibration curves and control charts were performed using Excel (Microsoft Office, Redmond, WA, USA). All the multivariate data analysis, including principal component analysis (PCA), hierarchical cluster analysis (HCA), correlation matrix analysis (CMA), partial least-square discriminant analysis (PLS-DA), as well as receiver operating characteristic (ROC) curves were processed using MetaboAnalyst 4.0 (wwww.metaboanalyst.ca) [32], where the data were normalized using a generalized *log*-transformation and autoscaling unless otherwise stated. PCA, HCA and CMA methods were used for data visualization (i.e., data trends, outlier detection), and comparing technical variance relative to the overall biological variance, whereas the PLS-DA was used for selecting significant serum metabolites associated with the PAD progression. Additionally, ROC curves were performed only for the top-ranked ratiometric serum metabolites that discriminated between IC and CLTI based on the area under the curve (*AUC*) [32]. Baseline characteristics of PAD participants, including the control (CON), IC, and CLTI sub-groups were compared using Fisher exact tests for categorical variables and an analysis of variance (ANOVA) for continuous variables in addition to an independent Student *t*-test for comparing the IC and CLTI sub-groups. A one-way ANOVA was used to identify the significant differences between the CON, IC, and CLTI groups for all the metabolites with a polynomial contrasts analysis to identify the linear trends associated with disease progression and Welch’s F employed in the case of inequality of variance tested by Levene’s homogeneity test. This was followed by planned contrasts with contrast 1 comparing CON to all the PAD cases [IC + CLTI], as well as contrast 2 comparing IC to CLTI (i.e., clinical PAD sub-groups), which was further confirmed by *post-hoc* analyses with Gabriel’s and Games–Howell procedures. In addition, a two-tailed Student’s *t*-test was employed on non-transformed and *log*-transformed data to compare the CLTI to IC separately in the subgroup analysis while applying Benjamini Hochberg false discovery rate (FDR) correction (*q* < 0.05) for multiple hypothesis testing. Pearson correlations were calculated to evaluate the associations between the ABI and the metabolites on non-transformed data for the majority of variables and *log*-transformed data for non-normally distributed variables which was further confirmed with partial correlations adjusting for the BMI and smoking status. Normality tests, a Shapiro–Wilk test (*p* < 0.05), Pearson and partial correlations, ANOVA, *t*-tests and nonparametric statistical analysis (Kruskal–Wallis and Mann–Whitney U test) were performed using the Statistical Package for the Social Science (IBM SPSS, version 18.0, Armonk, NY, USA). MedCalc version 12.5.0 (MedCalc Software, Ostend, Belgium) was used for the generation of boxplots, as well as Bland–Altman % difference plot and a Passing–Bablok regression used for the inter-laboratory method comparison of serum creatinine concentrations.

## 3. Results

### 3.1. Cohort Demographics and Clinical Characteristics

This study comprised a cohort of non-diabetic older persons (*n* = 58) with an mean age of 63 years, including PAD patients at different stages of disease progression as clinically defined by the six-stage Rutherford classification (stages 1–3: IC, and stage ≥ 4: CLTI), as well as non-PAD controls (CON). A summary of the study demographics and the other clinical characteristics for the participants is summarized in Table 1. There were no significant differences in age, sex, body mass index (BMI), glycated hemoglobin, as well as leukocyte and platelet counts between the three patient sub-groups. The IC and CLTI patients presented with a higher incidence of dyslipidemia (>80%), hypertension (>65%), coronary artery disease (>40%), statin/antiplatelet medication use (>80%), and notably smoking (~95%) than the controls. However, all patients with a 6-month history of acute coronary syndrome, heart failure, or uncontrolled arrhythmia were excluded in this study eliminating active coronary symptoms at the time of blood sampling. Importantly, when analyzing the differences between the two PAD subgroups, IC and CLTI, the patients were closely matched with no statistical differences (*p* > 0.05) in anthropometric properties, comorbidities, or medication use with the exception for ABI (<0.90; *p* = 2.36 × 10^−9^), which was lower in the CLTI (mean ABI = 0.38) as compared to the IC group (mean ABI = 0.57) and non-PAD controls (mean ABI = 1.08). Moreover, there were only two PAD patients diagnosed with renal insufficiency (1 CLTI; 1 IC), thus the vast majority of participants had normal kidney function at the time of recruitment.

### 3.2. The Serum Metabolome of PAD Patients

**Figure 1:**
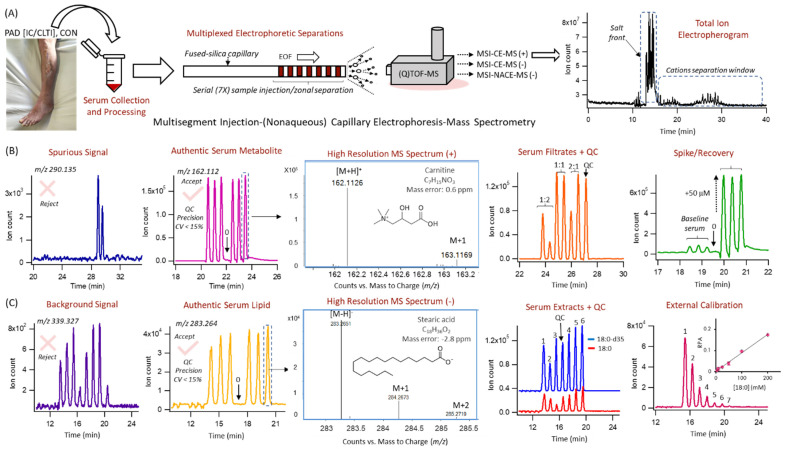
(**A**) Nontargeted metabolite profiling of serum samples from PAD patients using MSI–(NA)CE–MS under three different configurations, where the black trace depicts a total electropherogram using aqueous BGE conditions with positive mode detection. This multiplexed separation method relies on a serial injection of 6 serum samples and a quality control (QC) within each run to enhance sample throughput and data fidelity when using temporal signal pattern recognition. A rigorous data-filtering process allows for the reliable authentication of metabolites based on their accurate mass (*m/z*), which are measured in 6 replicate serum samples with acceptable precision (CV < 15%) with no background signal in blank (0) as shown for (**B**) carnitine and (**C**) stearic acid (18:0) under positive and negative ion mode detection, respectively. Various serial injection configurations are illustrated, such as a replicate injection of QC samples with a blank extract to filter out spurious and background signals in ESI–MS, the assessment of technical precision and potential sample carry-over for authentic serum metabolites, a randomized analysis of 6 serum samples from individual PAD patients along with a QC, a spike-recovery study to evaluate accuracy and confirm the identity based on co-migration, and a 7-point calibration curve for serum metabolite quantification.

Nontargeted analysis of the serum metabolome was performed by MSI–CE–MS and MSI–NACE–MS under three configurations for polar/hydrophilic and non-polar/lipophilic metabolites, respectively. Each run comprised a serial injection of seven serum samples for the CON, IC and CLTI patient sub-groups that were randomly analyzed in duplicate (except for fatty acids that used a single analysis), together with a pooled serum sample as a QC, to monitor for the long-term signal drift as shown in Figure 1A. Serum metabolites were authenticated based on their characteristic accurate mass and relative migration time under positive or negative ion mode (*m/z*:RMT:mode) when using multiplexed separations together with temporal signal pattern recognition [33]. For instance, only serum metabolites measured with adequate precision (CV < 30%, *n* = 6) with no signal detected in blank filtrate/extract [26] were initially selected after rejecting spurious signals, background artifacts, as well as redundant ions derived from the same metabolite (e.g., in-source fragments, isotopic signals, and adducts) that constitute a majority of signals (>90%) in ESI–MS [34]. Additionally, the identity of most serum metabolites was confirmed by spiking with authentic standards (level 1) based on their co-migration (RMT < 1%) with low mass error (<5 ppm), which was also used for evaluating method accuracy (i.e., spike/recovery) (Figure 1B), and quantification using external calibration curves (Figure 1C). Otherwise, seven serum metabolites with unknown chemical structures were annotated based on their most likely molecular formula (level 4). Moreover, serum metabolites were reported only if they satisfied two additional inclusion criteria to reduce false discoveries in metabolomics, namely they were measured with adequate precision throughout the entire study (CV < 30% from QC runs) and detected with high frequency (>75%) in all serum samples. This iterative process of data filtering and selection culminated in a final data matrix of 85 serum metabolites reliably measured in the majority of the samples in this cohort, including 42 hydrophilic cations, 18 hydrophilic anions and 25 lipophilic anions (Table S1). Overall, the coverage of the serum metabolome when using three configurations in MSI-(NA)CE-MS, included a diverse range of circulating metabolites ranging from amino acids, amines, organic acids, long-chain fatty acids, ketone bodies, hexoses, and osmolytes/uremic toxins.

**Figure 2:**
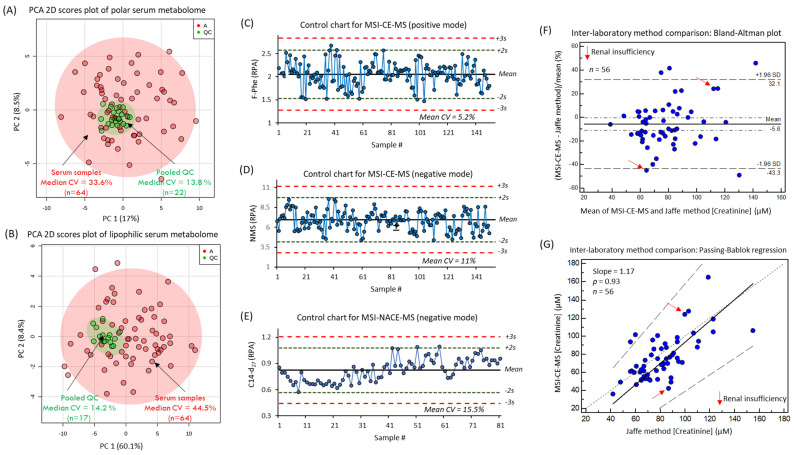
Two-dimensional scores plot from the principal component analysis (PCA) of the *glog*-transformed and autoscaled serum metabolome data used to compare the intersubject biological variance relative to the technical variance from the repeat analysis of pooled serum QC samples for (**A**) the hydrophilic serum metabolome and (**B**) the lipophilic serum metabolome. Control charts for (**C**) the recovery standard (F-Phe) measured under aqueous positive ion mode, for (**D**) the recovery standard 2-napthalenesulfonic acid (NMS) measured under aqueous negative ion mode, and for (**E**) the recovery standard 14-d27 measured under nonaqueous negative ion mode for all serum and QC samples demonstrate acceptable precision (*CV* = 5.2–15.5%) with no outliers exceeding the warning limits (±3 s). (**F**) Bland–Altman percent difference plot for comparing the mutual agreement between the serum creatinine concentrations measured independently by MSI–CE–MS and Jaffé colorimetric methods at two different laboratories from 56 participants. Overall, the data are randomly distributed with a modest mean bias of –5.6% with four outliers outside agreement limits. (**G**) A Passing–Bablok regression analysis demonstrates no significant deviation (dotted lines; 95% confidence interval) from the line of equality (*p* > 0.05) with a slope of 1.17 (black line; regression line).

As expected, the overall biological variance of the serum metabolome (median CV = 39%, *n* = 58) was considerably larger than the technical precision of the method based on the repeated analysis of a QC sample in each run (median CV = 14%, *n* = 17–22) as shown in the PCA 2D scores plots in Figure 2A,B. Moreover, control charts for the three recovery standards used in MSI–CE–MS (10 μM F-Phe and NMS) and MSI–NACE–MS (50 μM 14:0-d27) added to all serum samples, prior to ultrafiltration or MTBE extraction, further demonstrate the acceptable intermediate precision (mean CV < 15%) with few outliers (<2%, *n* = 57 total runs) exceeding warning limits (±2 s) (Figure 2C–E). An inter-laboratory method comparison of the serum creatinine concentrations measured independently from 56 participants was also performed by MSI–CE–MS relative to the Jaffé colorimetric method, which was used for the estimation of the glomerular filtration rate (GFR) for patients as an indicator of kidney function [20]. In this case, a Bland–Altman % difference plot confirms a good mutual agreement for serum creatinine determination by both methods with a mean bias of −5.6% (Figure 2F). In addition, there was a random distribution in the data with few outliers (4 of 56) exceeding the agreement limits (±2 s). Similarly, a Passing–Bablok regression analysis (Figure 2G) reveals no statistically significant difference from the line of equality (*p* = 0.93) with a modest positive slope of 1.17. Only two PAD patients (1 IC and 1 CLTI) out of the 58 study participants were diagnosed with renal insufficiency. Furthermore, no patients had mean serum creatinine concentrations exceeding a clinically relevant cutoff value (>150 μM) for older persons, that is indicative of renal failure [35] in the absence of urinary creatinine clearance for the calculation of the GFR.

### 3.3. Differentiating Serum Metabolites of PAD Progression

**Figure 3:**
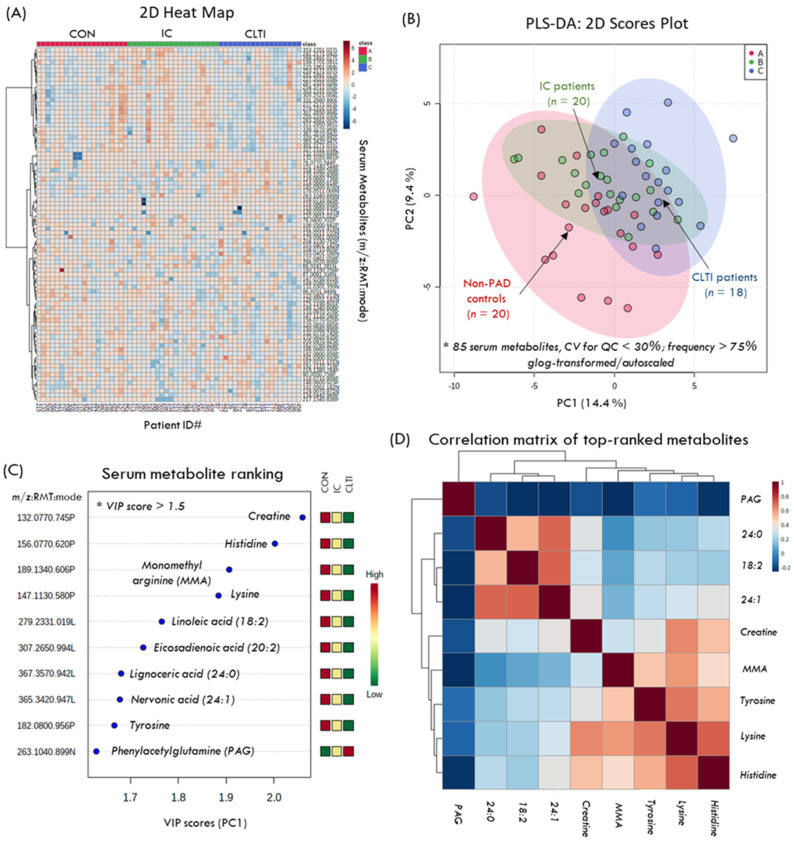
(**A**) Two-dimensional heat map of the serum metabolome of PAD patient sub-groups (IC, CLTI) and non-PAD controls (CON) that summarizes the overall data structure of this study. (**B**) Two-dimensional scores plot using a partial least-square discriminant analysis (PLS-DA) model to differentiate the metabolic phenotype of late-stage CLTI (*n* = 18) from early onset IC (*n* = 20) cases as compared to age and sex-matched CON (*n* = 20) based on 85 serum metabolites/lipids. (**C**) Ten top-ranked serum metabolites that differentiate PAD patients and non-PAD controls based on a variable importance in projection (VIP scores > 1.5). (**D**) Correlation matrix depicts two main clusters of circulating metabolites associated with PAD, including circulating amino acids/amines strongly correlated (*r*~0.70) to lysine (histidine, tyrosine, MMA: monomethylarginine, and creatine), as well as long-chain fatty acids (18:2, 20:2, 24:0, 24:1) unlike serum phenylacetylglutamine (PAG).

Figure 3A depicts a 2D heat map with HCA that summarizes the overall data structure involving 85 serum metabolites consistently measured in all 58 study participants (with only 0.7% missing values), the including CON, IC, and CLTI sub-groups. Figure 3B depicts a 2D scores plot when using PLS-DA for the differentiation of the serum metabolic phenotype in CLTI (*n* = 18) from the IC patients (*n* = 20), as well as CON (*n* = 20) based on *glog*-transformed and autoscaled data. Figure 3C summarizes the 10 top-ranked serum metabolites largely responsible for group separation along the first principal component (variable importance in projection, VIP > 1.5), including creatine, lysine (Lys), histidine (His), monomethylarginine (MMA), tyrosine (Tyr), phenylacetylglutamine (PAG), and several long-chain fatty acids from hydrolyzed lipids (18:2, 20:2, 23:0, 24:1). Figure 3D depicts a correlation matrix for the top-ranked serum metabolites associated with PAD progression, which highlights two major clusters of strongly co-linear serum metabolites not correlated to PAG, namely amino acids (*r >* 0.60 with Lys), and fatty acids (*r* > 0.70 with linoleic acid, 18:2*n*-6). Univariate statistical analysis was also performed to confirm the significance of the serum metabolites associated with PAD progression when using a one-way ANOVA with planned contrasts as summarized in Table 2. Overall, 14 serum metabolites were determined as significant (*p* < 0.05) when using a linear contrast analysis model across all three categories (i.e., CON–IC–CLTI), including 10 metabolites identified by the PLS-DA model, as well as four additional serum metabolites, namely oxo-proline (oxo-Pro), behenic acid (23:0), creatinine, and cystine. Moreover, the phenylalanine:tyrosine ratio (Phe/Tyr) was higher in CLTI as compared to IC, which is an indicator of inflammation in PAD [16]. Importantly, most serum metabolites exhibited a linear change in their concentrations (or RPAs) as a function of PAD status (*p* < 0.05). Overall, discrimination between the major patient sub-groups follows a linear trend where IC clusters in the middle between the CON and the more severe CLTI cases; these results are analogous to trends depicted in the PLS-DA model (Figure 3B).

**Figure 4:**
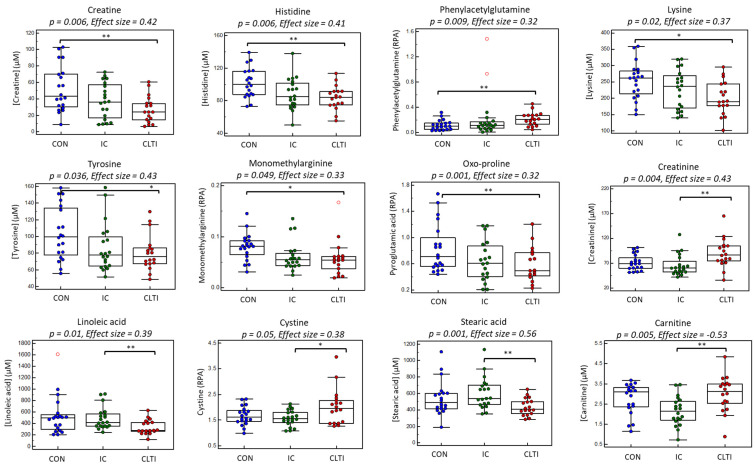
Box–whisker plots illustrating differences in the twelve top-ranked serum metabolites compared between the critical limb-threatening ischemia (CLTI) patients (*n* = 18), matched intermittent claudication (IC) patients (*n* = 20) and the non-PAD controls (*n* = 20). A one-way ANOVA test was performed to compare the means and identify significant changes in circulating metabolite concentrations between the three groups, as summarized in Table 2, where a polynomial contrasts analysis depicts most metabolites having a significant linear trend, proportional with disease progression. Planned contrasts were conducted by comparing non-PAD controls to PAD cases (IC + CLTI) (contrast 1; long bracket) followed by comparing IC to CLTI (contrast 2; short bracket) reflecting disease status and PAD progression, respectively where test significance is denoted as * *p* < 0.05 and ** *p* < 0.01. Serum stearic acid and carnitine were different between CLTI and IC when using an unpaired Student’s *t*-test after a FDR adjustment (*q* < 0.05). Serum metabolites responses in terms of absolute concentrations (μM), or relative peak area (RPA) if standards were not available.

Further analysis was next performed to identify the specific between-group differences without inflating type I error by using an ANOVA with two discrete planned contrasts, namely CON–PAD (contrast 1) and CLTI–IC (contrast 2). The box–whisker plots in Figure 4 show that circulating concentrations of creatine, His, oxo-Pro, Lys, Tyr and MMA were higher in non-PAD controls compared to PAD patients [IC + CLTI] unlike PAG (contrast 1, *p* < 0.05). Furthermore, serum creatinine, cystine, and Phe/Tyr were elevated in CLTI patients as compared to IC cases, whereas a series of circulating fatty acids (18:2, 22:0, 20:2, 24:0, 24:1) display the opposite trend. As expected, a similar outcome was found for serum creatinine independently measured by the Jaffé colorimetric method, confirming good mutual agreement with MSI–CE–MS results. An additional subgroup analysis using a two-tailed Student’s *t*-test was used to better evaluate changes associated with PAD progression since IC and CLTI patients were closely matched in terms of age, sex, BMI, smoking, co-morbidities, and medication use (Table 1). In this case, Table 3 summarizes 16 serum metabolites that were differentially expressed (*p* < 0.05) in the two PAD sub-groups, including 11 metabolites after a FDR adjustment (*q* < 0.05). In this case, serum creatinine, carnitine (C0), propionylcarnitine (C3), cystine, Phe/Tyr, and trimethylamine-*N*-oxide (TMAO) were elevated in CLTI as compared to IC cases, in contrast to several circulating fatty acids, including saturated/odd-chain fatty acids (15:0, 16:0, 17:0, 18:0). All putative serum biomarkers of PAD progression were also correlated with the ABI, notably stearic acid (18:0, *r* = 0.51, *p* = 0.001), as well as C0 and cystine (*r* = −0.48, *p* = 0.002). 

**Figure 5:**
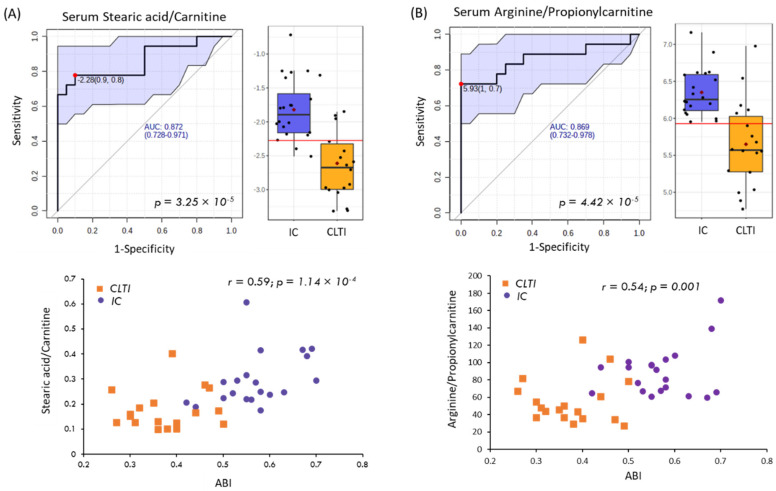
Upper panels show the receiver operating characteristic (ROC) curves and their corresponding box–whisker plots for the two top-ranked serum biomarker ratios used for discriminating chronic limb-threatening ischemia (CLTI, *n* = 18) from intermittent claudication (IC, *n* = 20) patients, including (**A**) stearic acid/carnitine (18:0/C0) and (**B**) arginine/propionylcarnitine (Arg/C3). Ratiometric ROC curves depict the area under the curve (*AUC*) and their 95% confidence intervals (blue shaded area). Lower panels depict the linear relationship of the serum biomarkers of PAD disease progression as a function of abnormal ankle–brachial index (ABI < 0.90) measurements with moderately strong Pearson correlation coefficients (*r* > 0.50; *p* < 0.002).

Lastly, receiver operating characteristic (ROC) curve analysis was also performed on all the serum metabolites and their ratios to demonstrate the reliable discrimination of severe CLTI from lower risk IC patients. Figure 5 shows two top-ranked ratiometric biomarkers in serum with an area under the curve or *AUC*~0.87 along with their 95% confidence intervals (0.73–0.98), namely 18:0/C0 and arginine (Arg)/C3. These two ratiometric biomarkers also exhibited a strong linear correlation with the ABI from PAD patients (*r* = 0.54–0.59, *p* < 0.001, *n* = 38). This is relevant for biomarker discovery in pilot studies to anchor aberrant metabolism to a validated physiological measure used for risk stratification of symptomatic PAD patients, which reflects increasing blockage of peripheral blood flow to the lower limbs.

## 4. Discussion

The lack of PAD awareness among physicians continues to pose a major diagnostic challenge due to its variable clinical manifestations and unpredictable aggressive progression. An improved screening strategy for the early detection of PAD in asymptomatic patients is required, given the poor sensitivity of specialized Doppler methods for ABI assessment at early stages of atherosclerosis in peripheral tissue [36]. In this case, prognostic biomarkers will augment well established traditional risk factors (e.g., smoking, diabetes, age, hyperlipidemia, renal dysfunction) while allowing for the reliable diagnosis of PAD, especially in high-risk patients with calcified vascular tissue not suitable for the ABI. Regrettably, blood-based protein biomarkers (e.g., inflammatory cytokines, C-reactive protein) have yet to be clinically validated for the routine screening of PAD, predict disease progression, and/or monitor the treatment responses of patients [6]. In this pilot study, we identified a panel of serum metabolites associated with PAD progression, which is important given the poor survivorship of patients with CLTI following invasive surgical interventions, including revascularization procedures and limb loss from amputation [2].

Untargeted metabolite profiling was performed on fasting serum samples collected from PAD patients, including well matched CLTI and IC cases, as well as non-PAD controls. Overall, 85 serum metabolites were consistently detected in most serum samples with good technical precision when using three different configurations in MSI–(NA)CE–MS. This multiplexed separation method takes advantage of unique data workflows and iterative data filtering processes to authenticate metabolites while also applying stringent QC to reduce false discoveries. We identified a panel of serum metabolites that differentiated CLTI from IC patients, as well as PAD from non-PAD controls. Serum creatine was found to be one of the most significant metabolites lower in PAD as compared to CON (*F* = 6.0, *p* = 0.006, effect size = 0.42), which also exhibited a linear change in concentration (*p* = 0.002) across the three patient sub-groups. Creatine is derived from dietary protein, as well as endogenously produced in the liver that is actively transported within muscle tissue, where it accumulates against a large concentration gradient. Creatine plays a key role in energy metabolism within skeletal muscle tissue by conversion into phosphocreatine via creatine kinase, which is an abundant phosphagen required for ATP regeneration during active muscle contractions [37]. As a result, lower serum creatine concentrations in CLTI likely reflects inadequate muscle energy storage and a loss of function with more advanced stages of PAD progression [22].

Additionally, lower antioxidant capacity within ischemic muscle tissue likely plays a role in the 0.83-fold reduction of serum His concentrations in PAD as compared to CON (*F* = 5.5, *p* = 0.006, effect size = 0.41). The antioxidant and anti-inflammatory properties of His, whether in free, peptide or protein form (e.g., intramuscular carnosine), have been attributed to the capacity of the imidazole ring to scavenge hydroxyl radicals and singlet oxygen species [38]. Our findings suggest that lower circulating His may contribute to a greater susceptibility to oxidative stress in PAD in accordance with reports of histidine protection of cardiac and vascular tissue injury [16,38]. Similar to trends identified in metabolite trajectories for creatine and His, PAD patients also exhibited a decrease in serum Lys, MMA and oxo-Pro concentrations relative to non-PAD controls (Table 2, Figure 3 and Figure 4). For example, the essential amino acid Lys acts as an exogenous inhibitor of plasmin-induced proteolysis that contributes to matrix remodeling, continued connective tissue degradation in the vascular wall and the formation of atherosclerotic lesions [39]. Since oxo-Pro is a degradation product of the major intracellular antioxidant glutathione [22,40], the 0.63-fold decrease in serum oxo-Pro in PAD cases as compared to non-PAD controls may indicate increased glutathione depletion and the activation of the glutathione salvage pathway that is a hallmark of deleterious oxidative stress [41,42]. This is consistent with elevated serum cystine in CTLI as compared to IC (*p* = 0.014), which is a biomarker of systemic oxidative stress prevalent in PAD, and is associated with adverse clinical outcomes independent yet synergistic to inflammation [43]. An opposing trend was found for serum PAG with a 1.9-fold higher concentration in PAD as compared to CON (*F* = 5.4, *p* = 0.009, effect size = 0.32). This circulatory uremic toxin is an independent risk factor for cardiovascular disease, where elevated serum PAG may serve as a predictor of overall mortality in high-risk patients with chronic kidney disease [44], but it has not been reported in PAD patients without renal dysfunction as was the case for most participants (>96%) in our study.

Plasma metabolomic profiles of near-term death PAD patients was previously reported to be strongly correlated with lipid metabolism based on differentiating chemical signals from fatty acid acyl chain protons in lipoprotein species [14]. However, as this study used NMR, the exact identity of fatty acids associated with PAD progression was not fully elucidated. Our results confirmed aberrant circulating lipid metabolism in terms of several serum fatty acids (total hydrolyzed) that were highly co-linear (Figure 3D) and consistently reduced in PAD patients relative to CON participants (Table 2), notably when comparing CLTI to IC cases in PAD patient sub-groups (Table 3). The essential omega-6 fatty acid, linoleic acid (18:2*n*-6), derived largely from dietary vegetable oils and nuts, has been the subject of conflicting reports regarding its putative pro- or anti-inflammatory roles [45]. A recent meta-analysis of 30 cohort studies supports that higher blood or adipose tissue levels of 18:2*n*-6 are associated with lower, and not higher, risk of cardiovascular disease, in agreement with our results [46]. Besides their roles in cell membrane phospholipid structure and energy metabolism, polyunsaturated fatty acids also serve as precursors of bioactive mediators of inflammation that have also been implicated in PAD pathophysiology [47]. For example, a randomized clinical trial involving 7435 participants reported that a Mediterranean diet supplemented with either extra-virgin olive oil or nuts was associated with a lower risk of PAD incidence as compared to a low-fat counseled control group [48]. Similarly, odd-chain/saturated fatty acids, 15:0 and 17:0, have been associated with lower ischemic heart disease and type 2 diabetes incidence [49], which are derived from dietary intake of full-fat dairy, as well as fiber and gut microflora activity [24]. Overall, serum 18:0 was the most significantly depleted in CLTI as compared to IC patients after a FDR adjustment (*q* < 0.05), that was also moderately correlated with the ABI (*r* = 0.51, *p* = 0.01). Mounting evidence supports that increasing circulating 18:0 lipids are associated with reduced blood pressure and improved heart function, which also promotes mitochondrial fusion with greater beta-oxidation activity [50] in late-stage PAD patients with severe muscle ischemia. Further work is warranted to confirm the exact role of serum fatty acids that are depleted with PAD progression, which interestingly did not include omega-3 fatty acids, such as eicosapentaenoic acid (20:5*n*-3) and docosahexaenoic acid (22:6*n*-3) that are associated with dietary intake of oily fish [24].

**Figure 6:**
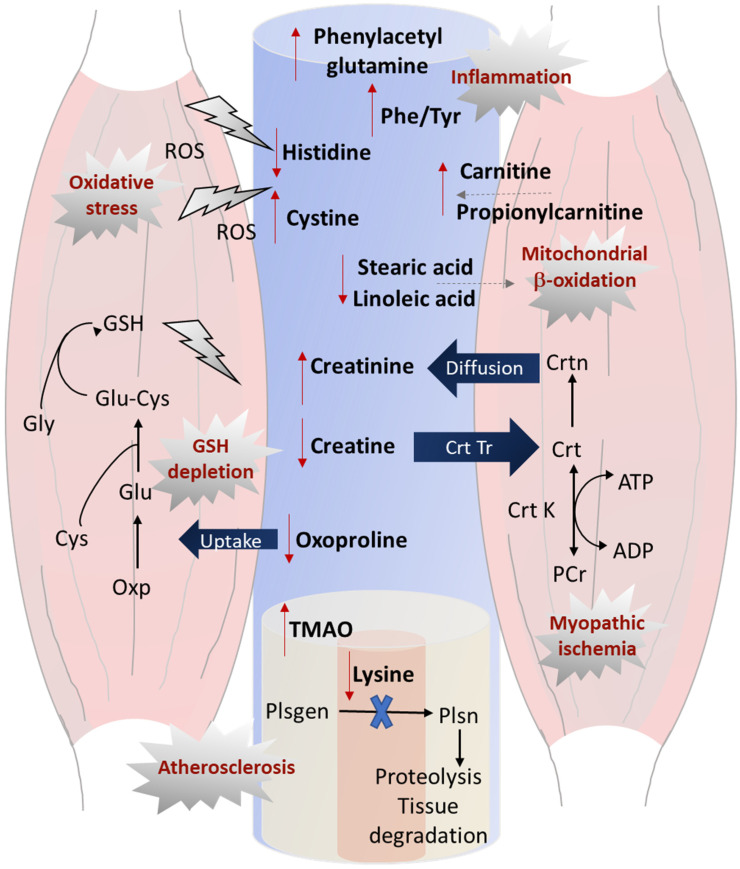
Schematic illustrating the systemic effects of an aberrant circulatory metabolism reflecting PAD progression in serum that also reflects the localized ischemia of skeletal muscle in lower limbs. Reduced serum lysine, an inhibitor of plasmin activation, in pathological states, leads to excessive proteolysis and vascular tissue degradation that is exacerbated by elevated TMAO, further promoting atherosclerosis in CLTI. Serum oxoproline, a key precursor used in the glutathione (GSH) salvage pathway, decreases in circulation as it is transported within the muscle to support intracellular glutathione recycling in response to elevated oxidative stress as reflected by lower histidine, and higher cystine in serum. Reduced creatine availability leads to lower intramuscular phosphocreatine, attributing to myopathic ischemia and perturbed energy homeostasis within the muscle that coincides with elevated creatinine concentrations in serum for CLTI. Impaired mitochondrial beta-oxidation is reflected by higher circulating carnitine and propionylcarnitine, that are not stored within ischemic muscle tissue in conjunction with lower circulating lipids/fatty acids, such as stearic acid and linoleic acid. Lastly, increased serum phenylacetylglutamine and Phe/Tyr reflect increased inflammation in CLTI, which are risk factors for all-causes mortality and cardiovascular disease. Abbreviations correspond to ROS: reactive oxygen species, ADP: adenosine diphosphate, ATP: adenosine triphosphate, Crt K: creatine kinase, Crt Tr: creatine transporter, Plsgen: plasminogen, Plsn: plasmin.

Due to potential confounding when comparing PAD patients to non-PAD controls, several serum metabolites were also differentially expressed (most satisfying a FDR adjustment, *q* < 0.05) in well matched CI and CLTI patients (Table 3). A mean 1.3-fold increase in serum carnitine (*p* = 0.005) likely reflects severe myofiber degeneration occurring in CLTI as compared to a less acute muscle atrophy in IC due to its key role in mediating the transport of long-chain fatty acids within the mitochondria for beta-oxidation [51]. Moreover, TMAO generated from dietary phosphatidylcholine and carnitine via the action of gut microbiota has been widely reported as a pro-atherogenic metabolite in CAD, and circulating TMAO predicts higher risk of 5 year all-cause PAD mortality [52,53]. A higher degree of atherosclerosis associated with CLTI, as compared to IC, is consistent with elevated serum TMAO concentrations (*p* = 0.019) in the PAD sub-group analysis as reflected by their lower ABI measurements (Table 1). In addition, creatine deficiency in circulation indicates a state of a diminished energy supply among CLTI patients, possibly contributing to disease progression. Creatine undergoes spontaneous irreversible non-enzymatic degradation within skeletal muscle tissue into creatinine, which diffuses out of the muscle tissue into the circulation to be excreted by glomerular filtration through the kidneys. In our study, a mean 1.3-fold increase in serum creatinine was measured in CLTI as compared to IC patients (*p* = 0.004), which was replicated independently on two different instrumental platforms/laboratories when using the Jaffé colorimetric assay and MSI–CE–MS (Figure 1G; Table 2). This finding highlights the consequence of ischemic muscle with advanced CLTI contributing to elevated serum creatinine, which was reported to predict mortality in PAD patients with renal failure independent of hypertension and type 2 diabetes [54]. An obvious confounder would be impaired renal function in CLTI, but our IC and CLTI patients were well balanced with only one participant from each group diagnosed with renal insufficiency. Furthermore, no other known differences exist among major PAD sub-groups, including comorbidities, smoking and medication use, with the exception of the ABI and clinical symptoms (i.e., leg pain and reduced mobility). Lastly, we observed a higher serum Phe/Tyr in CLTI relative to IC patients, consistent with recent reports of this as a ratiometric biomarker of inflammation in advanced stages of PAD [16], as well as acute ischemic stroke that is also correlated with serum cytokines, such as interleukin-6 [55]. Given the need for the improved risk stratification of PAD, other ratiometric biomarkers were found to be superior to Phe/Tyr (*AUC* = 0.708, *p* = 0.0216) as a diagnostic biomarker of CLTI in our study. For instance, the ratio of serum 18:0/C0 and Arg/C3 demonstrated better accuracy in differentiating CLTI from IC patients, with an *AUC*~0.87 (*p* = 4.0 × 10^−5^) from the ROC curves (Figure 5). As expected, these ratiometric serum biomarkers were also strongly correlated (*r* = 0.54−0.59, *p* < 0.001) with the ABI and may thus prove useful for routine monitoring PAD progression and/or allow for the early detection of PAD in asymptomatic patients in a primary care setting. Since PAD is a complex clinical syndrome contributing to peripheral ischemia and claudication, Figure 6 provides a systemic overview of the serum metabolites identified in this study that reflect its underlying pathophysiology, including elevated oxidative stress, inflammation, glutathione depletion, and perturbed energy homeostasis that contribute to severe ischemic muscle dysfunction. This panel of serum metabolites offers a convenient approach for diagnostic testing complementary to the ABI, that may also predict CLTI onset, while guiding optimal dietary, pharmacological and/or surgical interventions to mitigate PAD progression relevant to improving long-term patient survivorship and their quality of life.

## 5. Conclusions

In summary, our work has revealed distinctive metabolic signatures associated with PAD progression in age/sex-matched non-diabetic patients, largely without renal failure or differences in prescribed medications (e.g., statins, antiplatelets). We applied a high throughput metabolomics platform for the nontargeted analysis of circulating metabolites and hydrolyzed lipids from fasting serum samples, while applying a rigorous approach to data filtering to reduce the false discoveries with good technical precision. Certain serum metabolites have been reported in previous targeted and nontargeted metabolomic studies as indicative of PAD, using different instrumental platforms (NMR, GC–MS, LC–MS) that further corroborates our findings, including His, C0, TMAO, creatinine, Tyr and Phe/Tyr [14,15,16,17,52,53,54]; however, we did not observe differences in central energy metabolites (e.g., glutamate, 3-hydroxybuyrate, ketoglutarate) and metabolites from the urea cycle (e.g., ornithine, citrulline) that were also measured by MSI–CE–MS with stringent QC. In fact, discordant results were reported in two studies as related to the role of the urea cycle, likely reflecting potential confounding due to comorbidities in recruited PAD patients [15,16]. Importantly, we report for the first time that lower serum creatine, MMA, oxo-Pro, cystine, and several long-chain fatty acids are indicative of CLTI as compared to IC and/or non-PAD controls, with opposing trends for the uremic toxin, PAG. Overall, serum 18:0/C0 and Arg/C3 were among the most promising ratiometric biomarkers of PAD to accurately differentiate CLTI from IC, which also had strong positive correlations to the ABI. This panel of serum metabolites were related to multiple pathological processes associated with the clinical syndrome of PAD, including muscle energy perturbations, vascular remodeling, atherosclerosis, myopathic ischemia, inflammation, and oxidative stress. Major limitations to our pilot study include the modest sample size of PAD patients recruited from a single center, which did not include patient diet records to assess the influence of habitual diet on PAD progression. Future work is needed to better validate the clinical utility of lead serum metabolites as potential diagnostic, prognostic and/or treatment biomarkers of PAD in prospective studies involving larger patient cohorts, including monitoring responses following vascularization surgery. Moreover, muscle metabolomic studies [37] may prove useful to better understand the deleterious impacts of chronic muscle ischemia in localized tissues, as related to the pathophysiology of PAD when compared to systemic changes of metabolism in serum. The specificity of PAD biomarkers also needs to be evaluated as compared to other related cardiometabolic diseases, including CAD, type 2 diabetes, chronic kidney disease, as well as sarcopenia in older persons [22].

## Figures and Tables

**Table 1:** Baseline patient demographics and clinical characteristics for two peripheral artery disease (PAD) sub-groups (intermittent claudication (IC), chronic limb-threatening ischemia (CLTI)) and non-PAD controls (CON).

Parameter	CON (*n* = 20)	IC (*n* = 20)	CLTI (*n* = 18)	*p*-Value
Rutherford stage	-	1–3 (2.75 ± 0.4)	≥4 (4.11 ± 0.3)	-
Walking distance (m)	>1000	530	<160	-
ABI	1.08 ± 0.09	0.57 ± 0.08	0.38 ± 0.07	3.06 × 10^−33^; 2.36 × 10^−9^
Age (years)	62.6 ± 6.6	61.0 ± 7.4	65.2 ± 5.6	0.151; 0.055
BMI (kg/m^2^)	26.6 ± 2.5	24.3 ± 3.0	24.9 ± 3.6	0.061; 0.631
HbA1c (%)	5.75 ± 0.51	5.98 ± 0.50	5.58 ± 0.99	0.217; 0.124
Leukocytes	6.6 ± 2.2	7.8 ± 2.5	8.4 ± 3.4	0.156; 0.534
Platelets	251 ± 76	244 ± 64	209 ± 65	0.152; 0.118
Males (%)	50 (10/20)	55 (11/20)	72 (13/18)	0.401; 0.224
Smoking (%)	55 (11/20)	95 (19/20)	94 (17/18)	0.002; 0.730
Diabetes mellitus (%)	0	0	0	-
Hypertension (%)	40 (8/20)	65 (13/20)	72 (13/18)	0.099; 0.450
Hyperlipidemia (%)	39 (7/20)	85 (17/20)	83 (15/18)	0.001; 0.616
Renal insufficiency (%)	0 (0/20)	5 (1/20)	6 (1/18)	0.76; 0.730
Coronary artery disease (%)	0 (0/20)	40 (8/20)	61 (11/18)	0.001; 0.165
Statin use (%)	30 (6/20)	80 (16/20)	100 (18/18)	<0.001; 0.066
Antiplatelet use (%)	50 (10/20)	100 (20/20)	100 (18/18)	<0.001; -

Data shown as the mean ± standard deviation for continuous variables and % (number of cases/total) for categorical variables. *p*-value represents the overall difference between the three groups where significant differences are observed for (*p* < 0.05) calculated Fisher exact tests for categorical variables and ANOVA for continuous variables; followed by *p*-value for PAD subgroup comparison between CLTI and IC calculated using independent samples Student’s *t*-test or non-parametric Mann–Whitney U test (only for body mass index, BMI). Smoking status reflects numbers of past/current smokers.

**Table 2:** Top-ranked serum metabolites showing significant changes reflecting disease progression when comparing PAD cases to non-PAD controls, as well as IC to CLTI patient sub-groups when using a one-way ANOVA with planned contrasts, including their correlation to the ABI.

Metabolite ID	*m/z*:RMT:mode	*F*-Value	*p*-Value Overall	*p*-Value Linear ^a^	Effect Size ^b^	*p*-Value Contrast 1 PAD:CON ^c^	FC PAD:CON ^c^	*p*-Value Contrast 2 CLTI:IC ^d^	FC CLTI:IC ^d^	*r* Correlation to ABI ^e^	*p*-Value for *r*
Creatine, HMDB000064	132.077:0.745:p	6.02 *	0.006	0.002	0.422	0.008	0.65	0.097	0.75	0.44	0.001
Histidine, HMDB000117	156.077:0.620:p	5.54	0.006	0.003	0.410	0.002	0.85	0.435	0.95	0.38	0.004
Phenylacetylglutamine, HMDB0006344	263.104:0.899:n	5.43 *	0.009	0.017	0.319	0.030	1.89	0.880	0.94	−0.30	0.020 ^f^
Lysine, HMDB0000182	147.113:0.580:p	4.23	0.020	0.005	0.365	0.014	0.85	0.137	0.88	0.35	0.007 ^f^
Tyrosine, HMDB0000158	182.080:0.9564:p	3.53	0.036	0.014	0.338	0.012	0.81	0.520	0.94	0.34	0.008 ^f^
Monomethylarginine, HMDB0029416	189.134:0.606:p	3.19	0.049	0.022	0.332	0.005	0.74	0.378	0.93	0.32	0.014 ^f^
Oxo-proline, HMDB0000267	128.035:1.137:n	2.89	0.054	0.028	0.316	0.021	0.69	0.638	0.86	0.34	0.013
Creatinine	Jaffé method	6.57	0.003	0.002	0.446	0.055	1.27	0.003	1.25	−0.31	0.020
Creatinine, HMDB0000562	114.066:0.614:p	6.14	0.004	0.011	0.428	0.271	1.07	0.001	1.30	−0.30	0.035
Linoleic acid (18:2*n*-6), HMDB0000673	279.233:1.0189:l	4.96	0.010	0.007	0.390	0.101	0.78	0.009	0.95	0.24	0.066
Eicosadienoic acid (20:2), HMDB0005060	307.265:0.994:l	4.30	0.018	0.010	0.368	0.089	0.79	0.019	1	0.25	0.061
Nervonic acid (24:1), HMDB0002368	365.342:0.947:l	3.96	0.025	0.012	0.356	0.095	0.86	0.026	0.75	0.23	0.085
Phenylalanine/Tyrosine	-	3.67	0.032	0.026	0.343	0.22	1.10	0.018	1.15	−0.25	0.055
Behenic acid (22:0), HMDB0000944	339.327:0.969:l	3.49	0.038	0.026	0.336	0.187	0.91	0.024	0.75	0.22	0.105
Lignoceric acid (23:0), HMDB0002003	367.358:0.942:l	3.45	0.037	0.015	0.334	0.088	0.84	0.045	0.75	0.26	0.050
Cystine, HMDB0000574	241.030:0.933:p	3.15 *	0.050	0.028	0.377	0.305	1.07	0.019	1.29	−0.24	0.065

^a^*p*-value for a linear trend when applying polynomial contrasts analysis; ^b^ Effect size calculated based on eta-squared; ^c^ *p*-value and mean FC for planned contrast 1 comparing PAD to CON; ^d^ *p*-value and mean FC for planned contrast 2 comparing CLTI to IC; ^e^ Pearson correlation (*r*) on normally-distributed non-transformed or *log*-transformed serum metabolites to the ABI, after adjusting for BMI and smoking. ^f^ not significant after adjusting for BMI and smoking. * Welch’s *F*-test employed in case of inequality of variance tested by Levene’s homogeneity test. Abbreviations correspond to RMT: relative migration time, p: positive aqueous mode, n: negative aqueous mode, l: negative nonaqueous mode, CON: non-PAD controls, PAD: peripheral artery disease [IC + CLTI], CLTI: critical limb-threatening ischemia, IC: matched intermittent claudication, FC: fold-change, ABI: ankle–brachial index.

**Table 3:** Top-ranked serum metabolites comparing IC (*n* = 20) to CLTI (*n* = 18) using the Student’s *t*-test and their correlation to ABI.

Metabolite ID	*m/z*:RMT:mode	*p*-Value	FDR *q*-Value ^a^	FC ^b^ (CLTI/IC)	*r* Correlation to ABI ^c^	*p*-Value for *r*
Stearic acid (18:0), HMDB0000827	283.264:1.005:l	0.001	0.014	0.72	0.51	0.001
Linoleic acid (18:2*n*-6) HMDB0000673	279.233:1.019:l	0.003	0.028	0.68	0.39	0.016
Heptadecanoic acid (17:0), HMDB0002259	269.249:1.030:l	0.003	0.029	0.72	0.43	0.007
Palmitic acid (16:0), HMDB0000220	255.233:1.030:l	0.004	0.030	0.73	0.37	0.024
Creatinine, HMDB0000562	114.066:0.614:p	0.004	0.031	1.30	−0.45	0.004
Carnitine, HMDB0000062	162.112:0.719:p	0.005	0.031	1.28	−0.48	0.002
Oleic acid; 18:1*n*-9 HMDB0000207	281.249:1.013:l	0.005	0.031	0.71	−0.04	0.756
Heptadecenoic acid (17:1*n*-9), HMDB0062437	267.233:1.026:l	0.008	0.043	0.73	−0.01	0.961
Propionylcarnitine, HMDB0000824	218.138:0.784:p	0.008	0.043	1.37	0.09	0.507
Eicosadienoic acid (20:2*n*-6), HMDB0005060	307.265:0.994:l	0.009	0.047	0.72	0.37	0.023
Pentadecanoic acid (15:0), HMDB0000826	241.217:1.042:l	0.010	0.047	0.66	0.33	0.044
Cystine, HMDB0000574	241.0299:0.933:p	0.014	0.061	1.29	−0.48	0.002
Arachidic acid (20:0*n*-3), HMDB0002212	311.296:0.981:l	0.015	0.061	0.68	0.39	0.015
Trimethylamine-*N*-oxide, HMDB0000925	76.077:0.544:p	0.019	0.080	1.60	−0.44	0.005
Nervonic acid (24:1), HMDB0002368	365.342:0.947:l	0.024	0.091	0.75	0.29	0.083
Phe/Tyr ratio	-	0.022	0.103	1.19	−0.33	0.041

Two-tailed exact *p*-value on the log-transformed serum metabolome data. ^a^ FDR correction for multiple hypothesis testing; ^b^ mean fold-change (FC) ratio when comparing the relative ion response ratio for each metabolite as a ratio of CLTI/IC; ^c^ Pearson correlation on the normally distributed non-transformed or *log*-transformed data.

## Supplementary Materials

The following are available online at https://www.mdpi.com/2077-0383/9/6/1877/s1, Table S1: Summary of serum metabolome of PAD patients, that summarizes all 85 serum metabolites and hydrolyzed lipids measured in this study, and an excel file containing serum metabolome data matrix measured for all PAD patients (IC, CLTI) and non-PAD controls for authenticated metabolites/lipids measured by MSI–(NA)CE–MS under three different configurations is also provided, including quality controls. All serum metabolites are annotated by their accurate mass and relative migration time (*m/z*:RMT) and name (if identified), where responses reflect their ion response ratio normalized to an internal standard. This excel file also contains deidentified patient demographic and clinical information.
