# Supplementary material for: Serum Metabolic Signatures of Chronic Limb-Threatening Ischemia in Patients with Peripheral Artery Disease
Source: J Clin Med. 2020 Jun 16;9(6):1877. doi: 10.3390/jcm9061877 (PMC7355749; doi:10.3390/jcm9061877)
Supplement: Supplementary file 1 [file jcm-09-01877-s001.zip › PAD-SI.pdf]

SUPPLEMETARY INFORMATION

Serum Metabolic Signatures of Chronic Limb-Threatening Ischemia in Patients with Peripheral Artery Disease

Sandi M. Azab,[1, 2] Abdelrahman Zamzam,[3] Muzammil H. Syed,[3] Rawand Abdin,[4] Mohammad Qadura[3] and Philip Britz-McKibbin[1*]

[1] *Department of Chemistry and Chemical Biology, McMaster University, Hamilton, ON, Canada*

[2] *Department of Pharmacognosy, Alexandria University, Alexandria, Egypt*

[3] *Department of Surgery, St. Michael's Hospital, Toronto, ON, Canada*

[4] *Department of Medicine, McMaster University, Hamilton, ON, Canada*

* Corresponding author: E-mail: britz@mcmaster.ca

Supplemental Information: Supplementary data file description, Table S1

SUPPLEMENTARY DATA FILE DESCRIPTION

Serum Metabolome Data Matrix and Deidentified Patient Data.

An supplementary excel file containing serum metabolome data matrix measured for all PAD patients (IC, CLTI) and non-PAD controls for authenticated metabolites/lipids measured by MSI-(NA)CE-MS under three different configurations is provided, including quality controls. All serum metabolites are annotated by their accurate mass and relative migration time (*m/z*:RMT) and name (if identified), where responses reflect their ion response ratio normalized to an internal standard. This excel file also contains deidentified patient demographic and clinical information from this pilot study for full data transparency.

Table S1. Summary of 85 serum metabolites detected in PAD patients that are annotated based on their accurate mass (*m/z*), relative migration time (RMT), ionization mode (l = non-aqueous negative mode, p = aqueous positive mode, n = aqueous negative mode), metabolite ID, most likely molecular formula, confidence level for identification, and technical precision of repeated QCs analyzed in each run.

***m/z*:RMT:mode**	**Metabolite ID**	**Molecular Formula**	**Confidence level**	**% CV**
Myristic-d27 acid (14:0-d27)	254.371:1.053:l	$C_{14}HD_{22}O_2$	1	6.0
Lauric acid (12:0)	199.170:1.081:l	$C_{12}H_{24}O_2$	1	14.2
Myristelaidic acid (14:1)	225.186:1.064:l	$C_{14}H_{26}O_2$	2	16.1
Myristic acid (14:0)	227.202:1.058:l	$C_{14}H_{28}O_2$	1	16.1
Pentadyclic acid (15:0)	241.217:1.042:l	$C_{15}H_{30}O_2$	1	29.2
Palmitoleic acid (16:1n7)	253.217:1.037:l	$C_{16}H_{30}O_2$	1	7.3
Palmitic acid (16:0)	255.233:1.030:l	$C_{16}H_{32}O_2$	1	11.6
Heptadecenoic acid (17:1n7)	267.233:1.026:l	$C_{17}H_{32}O_2$	2	14.5
Heptadecanoic acid (17:0)	269.249:1.017:l	$C_{17}H_{34}O_2$	1	12.0
Linolenic acid (18:3*n*-3)	277.217:1.023:l	$C_{18}H_{30}O_2$	1	6.8
Linoleic acid (18:2*n*-6)	279.233:1.019:l	$C_{18}H_{32}O_2$	1	5.2
Oleic acid (18:1*n*-9)	281.249:1.013:l	$C_{18}H_{34}O_2$	1	5.4
Stearic acid (18:0)	283.2676:1.0051:l	$C_{18}H_{36}O_2$	1	14.9
Eicosapentaenoic acid (20:5*n*-3)	301.217:1.031:l	$C_{20}H_{30}O_2$	1	4.9
Arachidonic acid (20:4*n*-6)	303.233:1.028:l	$C_{20}H_{32}O_2$	1	6.5
Dihomo-linolenic acid (20:3*n*-6)	305.252:1.004:l	$C_{20}H_{34}O_2$	2	7.8
Eicosadienoic acid (20:2)	307.265:0.994:l	$C_{20}H_{36}O_2$	2	7.6
Arachidic acid (C20:0)	311.295:0.981:l	$C_{20}H_{40}O_2$	1	27.1
Docosahexaenoic acid (22:6*n*-3)	327.233:1.034:l	$C_{22}H_{32}O_2$	1	6.3
Docosapentaenoic acid (22:5*n*-6)	329.253:0.992:l	$C_{22}H_{34}O_2$	2	14.3
Adrenic acid (22:4*n*-6)	331.268:0.990:l	$C_{22}H_{36}O_2$	1	6.2
Behenic acid (22:0)	339.327:0.969:l	$C_{22}H_{44}O_2$	1	21.8
Unknown#1	353.235:1.027:l	$C_{20}H_{34}O_5$	3	23.4
Nervonic acid (24:1*n*-9)	365.342:0.947:l	$C_{24}H_{46}O_2$	1	20.9
Lignoceric acid (24:0)	367.357:0.942:l	$C_{24}H_{48}O_2$	1	26.5
Glycine	76.040:0.702:p	$C_2H_5NO_2$	1	27.5
Trimethylamine-*N*-oxide	76.077:0.544:p	C_3H_9NO	1	19.4
Alanine	90.055:0.758:p	$C_3H_7NO_2$	1	11.7
γ-Aminobutyric acid	104.071:0.805:p	$C_4H_9NO_2$	1	18.0
Dimethylglycine	104.071:0.926:p	$C_4H_9NO_2$	1	24.2
Choline	104.108:0.569:p	$C_5H_{14}NO$	1	14.6
Serine	106.050:0.842:p	$C_3H_7NO_3$	1	7.2

Creatinine	114.066:0.614:p	$C_4H_7N_3O$	1	7.8
Proline	116.071:0.908:p	$C_5H_9NO_2$	1	6.3
Guanidoacetate	118.086:0.835:p	$C_3H_7N_3O_2$	1	17.0
Betaine	118.086:0.956:p	$C_5H_{11}NO_2$	1	11.9
Threonine	120.065:0.885:p	$C_4H_9NO_3$	1	6.7
Unknown#2	129.066:0.736:p	$C_5H_8N_2O_2$	4	7.5
Hydroxyproline	132.065:1.022:p	$C_5H_9NO_3$	1	6.4
Creatine	132.077:0.745:p	$C_4H_9N_3O_2$	2	8.2
Isoleucine	132.102:0.848:p	$C_6H_{13}NO_2$	1	8.6
Leucine	132.102:0.861:p	$C_6H_{13}NO_2$	1	7.5
Ornithine	133.097:0.578:p	$C_5H_{12}N_2O_2$	1	9.3
Unknown#3	134.044:0.969:p	$C_4H_7NO_4$	4	14.2
Hypoxanthine	137.046:1.058:p	$C_5H_4N_4O$	1	13.9
Glutamine	147.076:0.910:p	$C_5H_{10}N_2O_3$	1	5.5
Lysine	147.113:0.580:p	$C_6H_{14}N_2O_2$	1	6.6
Glutamic acid	148.060:0.923:p	$C_5H_9NO_4$	1	5.6
Methionine	150.058:0.896:p	$C_5H_{11}NO_2S$	1	5.3
TMAO (dimer)	151.144:0.544:p	C_3H_9NO	1	16.8
Histidine	156.077:0.620:p	$C_6H_9N_3O_2$	1	7.8
Unknown#4	160.133:0.709:p	$C_8H_{17}NO_2$	4	15.3
α-Aminoadipate	162.076:0.924:p	$C_6H_{11}NO_4$	1	12.9
Carnitine	162.112:0.719:p	$C_7H_{15}NO_3$	1	7.5
Phenylalanine	166.086:0.926:p	$C_9H_{11}NO_2$	1	6.7
Unknown #5	169.058:0.910:p	$C_5H_{10}N_2O_3$	4	25.5
Methylhistidine	170.092:0.635:p	$C_7H_{11}N_3O_2$	1	5.4
Arginine	175.119:0.601:p	$C_6H_{14}N_4O_2$	1	7.3
Citrulline	176.103:0.936:p	$C_6H_{13}N_3O_3$	1	5.3
Tyrosine	182.080:0.9564:p	$C_9H_{11}NO_3$	1	2.8
Monomethylarginine	189.134:0.606:p	$C_7H_{16}N_4O_2$	1	18.9
Acetylcarnitine	204.123:0.762:p	$C_9H_{17}NO_4$	1	7.5
Tryptophan	205.097:0.925:p	$C_{11}H_{12}N_2O_2$	1	7.4
Unknown#6	217.154:0.836:p	$C_{15}H_{20}O$	4	28.8
Propionylcarnitine	218.138:0.784:p	$C_{10}H_{19}NO_4$	1	13.5
Cystine	241.030:0.933:p	$C_6H_{12}N_2O_4S_2$	1	5.8
Cysteinylglycine disulfide	298.052:0.806:p	$C_8H_{15}N_3O_5S_2$	2	6.6
Pyruvic acid	87.009:1.338:n	$C_3H_4O_3$	1	26.9
Lactic acid	89.024:1.281:n	$C_3H_6O_3$	1	16.6
Phosphoric acid	96.970:1.949:n	H_3O_4P	2	14.7
Dimethylglycine	102.056:1.182:n	$C_4H_9NO_2$	1	25.5
3-Hydroxybutyric acid	103.040:1.142:n	$C_4H_8O_3$	1	29.8

2-Hydroxybutyric acid	103.040:1.168:n	$C_4H_8O_3$	1	23.4
α-Ketoisovaleric acid	115.040:1.213:n	$C_5H_8O_3$	1	30.9
Taurine	124.007:0.972:n	$C_2H_7NO_3S$	1	15.2
Pyroglutamic acid	128.035:1.137:n	$C_5H_7NO_3$	1	31.4
3-Methyl-2-oxovaleric acid	129.055:1.142:n	$C_6H_{10}O_3$	1	22.3
Aspartic acid	132.030:1.350:n	$C_4H_7NO_4$	1	22.4
Unknown#7	135.031:1.110:n	$C_4H_8O_5$	4	19.1
Uric acid	167.021:1.101:n	$C_5H_4N_4O_3$	1	16.3
Hippuric acid	178.051:1.009:n	$C_9H_9NO_3$	1	13.8
Glucose	179.056:1.000:n	$C_6H_{12}O_6$	1	16.1
Citric acid	191.020:2.418:n	$C_6H_8O_7$	1	26.1
Gluconic acid	195.050:0.976:n	$C_6H_{12}O_7$	1	26.6
Phenylacetylglutamine	263.104:0.899:n	$C_{13}H_{16}N_2O_4$	1	15.5
